# CAR-T Therapies in Solid Tumors: Opportunities and Challenges

**DOI:** 10.1007/s11912-023-01380-x

**Published:** 2023-02-28

**Authors:** Grace Guzman, Megan R. Reed, Kevin Bielamowicz, Brian Koss, Analiz Rodriguez

**Affiliations:** 1 Department of Neurosurgery, University of Arkansas for Medical Sciences, Little Rock, AR 72205 USA; 2 Department of Biochemistry and Molecular Biology, University of Arkansas for Medical Sciences, Little Rock, AR 72205 USA; 3 Division of Hematology/Oncology, Department of Pediatrics, Arkansas Children’s Research Institute, Little Rock, AR 72205 USA

**Keywords:** Immunotherapy, Chimeric antigen receptor T cell, Pediatric solid tumors

## Abstract

**Purpose of Review:**

This review will discuss the challenges facing chimeric antigen receptor (CAR)-T cell application for solid tumors and opportunities to overcome these obstacles. In addition, this review will examine therapies that are in development for pediatric solid tumors.

**Recent Findings:**

The similar success of CAR-T cell treatment for hematological malignancies has not been observed in solid tumors because of the hostile tumor microenvironment and tumor heterogeneity. Most strategies developed to combat these limitations emphasize combinatorial techniques that still require further testing. Preliminary results of multiple clinical trials, including GD2- and HER2-CAR-T cells, are encouraging but must be reproduced and validated on a larger scale.

**Summary:**

CAR-T cell application in solid tumors remains challenging, and most research is in development. Several clinical trials are ongoing for pediatric solid tumors. Early results are promising but demonstrate the need for CAR-T cell modification to prevent tumor recurrence.

## Introduction

Over the past several decades, impressive progress in multiple treatment modalities has dramatically improved the prognosis of pediatric cancer patients, with current overall survival (OS) of approximately 80% in the USA [1]. However, many pediatric cancer survivors endure severe chronic health conditions from toxicities associated with standard treatment of chemotherapy and radiation therapy (RT) [2]. In addition, improvements in mortality and survival have been limited in childhood cancers nervous system (CNS) gliomas, neuroblastomas, and sarcomas. Recent advances in cancer research have focused on novel targeted and immune-based therapies, including chimeric antigen receptor (CAR) T cells, to combat these limitations.

CAR-T cells are genetically engineered from patient- or donor-derived T cells that express synthetic receptors against specific tumor antigens (TA) to identify and target cancer cells [3–5]. CAR-T cells have four major components: an extracellular or antigen-recognition domain (usually single-chain variable fragments (scFv)), a hinge, a transmembrane, and an intracellular signaling domain (most commonly CD3ζ). Unlike conventional T cells, CARs function independently of the major histocompatibility complex (MHC), allowing for the recognition of a more extensive range of tumor-associated antigens [6•]. CAR design has evolved over four generations to increase the efficacy of the therapy. Second- and third-generation CARs include one or two co-stimulatory domains (often CD28 and/or 4-1BB) to enhance T cell proliferation, cytotoxicity, and survival. Fourth generation CARs, also known as T-cells redirected for universal cytokine-mediated killing (TRUCKS), are based on second-generation constructs with the addition of an inducible transgenic protein, such as a cytokine interleukin-12 (IL-12), to enhance anti-tumor activity [7]. Currently, the fifth generation of CARs is underway; the new CAR design is also based on second-generation constructs with the addition of a truncated cytoplasmic receptor and transcription factor (STAT3/5)–binding motif. Fifth-generation CARs have been shown to reactivate the immune system and maintain superior persistence compared to predecessors [8].

CAR-T therapy has shown monumental success in treating hematological malignancies. To date, the US Food and Drug Administration (FDA) has approved six CAR-T cell therapies indicated for hematological cancers, such as B-cell lymphoblastic leukemia and B-cell non-Hodgkin lymphomas [9]. Despite revolutionary progress in blood cancers, equivalent success has yet to be duplicated in solid tumor malignancies. Significant developments such as allogeneic or “off-the-shelf” CAR-T cells, which involve T cells from a donor's circulating blood, have attempted to improve success [10]. However, solid tumors present unique challenges, such as a hostile tumor microenvironment (TME) and heterogeneous antigen expression that hinder similar advancements [4, 11, 12]. In this review, we discuss the challenges that limit CAR-T development in solid tumors, review current pediatric CAR-T therapy clinical trials, and highlight proposed engineering mechanisms to make CAR-T cells a feasible option for solid tumors.

## Challenges to Successful CAR-T Cell Therapy in Solid Tumors

### Targeting Tumor Antigens and Tumor Heterogeneity

A central difference between solid tumors and hematological cancers is the intricacy in detecting a perfect target antigen. While target heterogeneity exists in blood cancers, these disorders more commonly tend to express individual markers (i.e., B-cell marker CD19) [13]. Additionally, solid tumors more often have tumor-associated antigens (TAA), where this antigen is highly expressed on the tumor itself but also at low levels in normal tissues [14]. Furthermore, solid tumors display TAA heterogeneity between tumor types (primary vs. metastatic) and patients with the same cancer [15, 16]. Consequently, a significant roadblock is designing a proper CAR-T cell with the capability of finding an ideal target antigen in solid tumors.

**Fig. 1:**
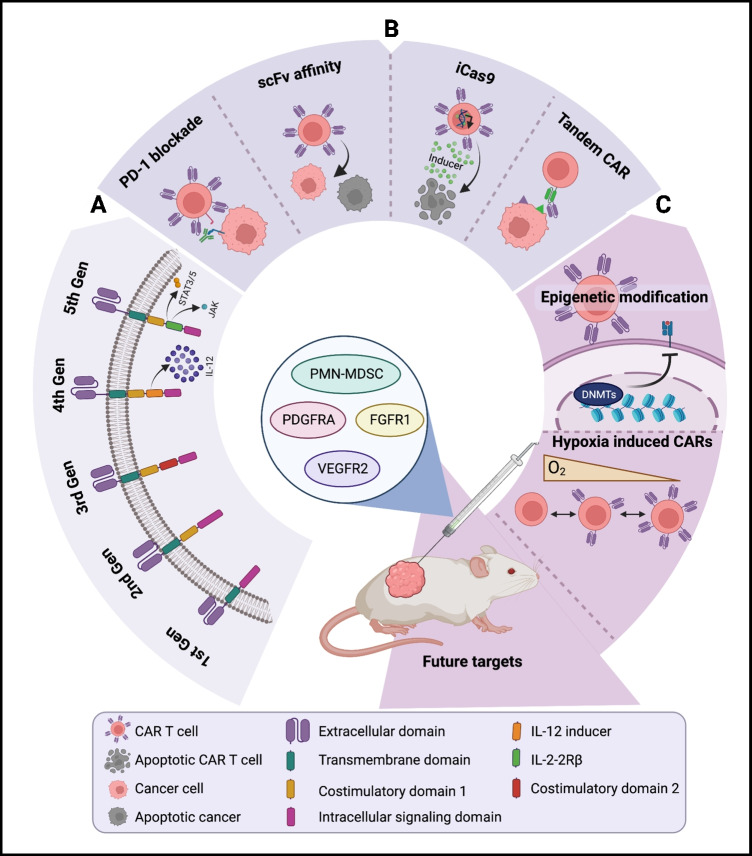
Progress of CAR-T cells in the treatment of solid neoplasms. (**a**) Evolution of CAR structure and function. (**b**) Current modifications of CAR-T cells to enhance or repress function. (**c**) Future directions of CAR-T cell treatment including novel methodologies and gene targets. Created with BioRender.com

Efforts to strengthen the ability of CAR-T cells to recognize ideal target antigens have focused on genomic and proteomic strategies (Fig. 1). Multi-specific CAR-T therapy, wherein two different CARs are introduced into the same T cell, is actively investigated and includes bicistronic CAR, tandem CARs (TanCAR), or LoopCAR methods [17, 18]. TanCAR constructs are composed of two scFv-binding domains joined in a linear fashion (aka tandem) with one exodomain; sometimes, it can contain a loop structure (LoopCAR). TanCARs are activated when either of the scFvs binds to a target antigen, prompting the cell to synchronously recognize two types of surface antigen and thus produce superior anti-tumor efficacy and decrease antigen escape [19]. Recently, a novel TanCAR targeting interleukin-13 receptor subunit alpha-2 (IL-13Rα2) and ephrin type-A receptor 2 (EphA2) was shown to be effective in both in vitro and in vivo glioblastoma (GBM) models. Muhammad et al. found that these TanCARs killed gliomas more efficiently than single-target CAR-T cells [20]. In addition, the TanCAR construct showed tremendous potential for preventing antigen escape and reducing off-target cytotoxicity compared to previously explored TanCAR for GBM consisting of IL-13 and HER2. A similar strategy to mitigate antigen loss involves multivalent CARs, in which multiple binding domains are included in the same CAR design. A multivalent CAR-T cell targeting HER2, GD2, and CD44v6 for breast cancer is in clinical trials (NCT04430595).

### Tumor Trafficking and Infiltration

Identifying an ideal target antigen is the first part of the battle; once a target antigen is found, the CAR-T cell must successfully reach the desired site and infiltrate the tumor. CAR-T cells are systemically administered for hematological malignancies and therefore have easy access to a target. In comparison, solid tumor CAR-T therapy must surmount several obstacles, such as the tumor stroma and abnormal vasculature that impede the penetration and homing of CAR-T cells [13].

Given that chemokines naturally regulate and stimulate immune cell migration, several studies have harnessed the chemokine system as a promising strategy to improve the activity of CAR-T cells [21]. Special attention has been directed to analyzing the expression profiles of chemokines in the TME of solid tumors to identify which chemokine axis is the best to exploit in different tumor types. In a hepatocellular carcinoma model, CXCR2-modified GDPC3 CAR-T cells significantly enhanced trafficking and anti-tumor efficacy compared to non-modified CAR-T and control T cells [22•]. Although the CXCR2 CAR-T cells displayed identical cytotoxicity as control groups, the cells exhibited accelerated migration ability in vitro. Similar encouraging outcomes of chemokine-modified CAR-T cells have been demonstrated in glioblastoma (CXCR1/CXCR2) and non-small-cell lung cancer (NSCLC) (CCR2B) xenograft models [23, 24]. While the modulation of chemokine axes shows great promise, their role in CAR-T therapy for solid tumors needs to be well defined, as chemokines also directly mediate tumor invasiveness and metastasis [25]. Currently, CXCR4-modified CAR-T treatment for multiple myeloma is in a clinical trial (NCT04727008).

Other developments include targeting fibroblast activation protein (FAP), an essential protease of cancer-associated fibroblasts (CAFs) that is highly overexpressed in many cancers, including lung, pancreatic, ovarian, and colorectal tumors [26]. FAP remodels the extracellular matrix (ECM) and promotes immunosuppression, thus limiting the ability of CAR-T cells to infiltrate the tumor. Some preclinical studies of FAP-CAR-T cells have been conducted for various solid cancers, such as lung and pancreatic cancers, showing increased homing and a more robust anti-tumor response [27]. Some of these studies also utilized cancer vaccines (e.g., HPV-E7) and immune checkpoint inhibitors (e.g., anti-CTLA 4) to increase inhibition of the immunosuppressive effects of FAP [28, 29]. Recently, the first-ever clinical trial of fourth-generation CAR-T targeting Nectin4/FAP in solid malignant tumors is underway (NCT03932565). This clinical trial aims to eliminate and prevent the recurrence of Nectin4/FAP-positive solid tumors through intratumoral injection of CAR-T cells. At the time of this publication, the trial was still in its recruitment phase.

### Hostile Tumor Microenvironment (TME)

Once a CAR-T cell invades the endothelial barrier, it must continue to survive and persist in the inhospitable TME. The TME presents a myriad of obstacles to CAR-T therapy, including the tumor stroma composed of suppressive cell types such as tumor-associated macrophages (TAMs) and myeloid-derived suppressor cells (MDSCs) and hypoxic conditions that hamper its execution [30]. Furthermore, there are differences in the composition of the TME between adult and pediatric solid tumors that may call for special considerations when developing therapies. For example, an immunogenic analysis of over 20 pediatric tumors showed significantly higher infiltration by CD68 + macrophages compared to adult carcinomas [31].

Extensive research has demonstrated that inhibiting immune checkpoint, programmed cell death protein-1 (PD-1), through PD-1 targeting monoclonal antibodies co-administrated with CAR-T infusion or PD-1 gene editing of CAR-T cells enhanced therapeutic efficacy in both preclinical and clinical models [32]. A clinical trial for NSCLC using MUC1-targeted CAR-T cells with PD-1 knockout (KO) through CRISPR-Cas9 determined the treatment to be safe, but success differed between patients (NCT03525782). Varied clinical outcomes using PD-1 blockade prompted efforts to seek other viable modifications to conquer the TME. One approach targeted transforming growth factor β (TGBβ), a critical multifunctional polypeptide that can hamper T cell effector function through various avenues like restricting the maturation of T helper cells [33]. One group used CRISPR/Cas9 to knock out TGF-β receptor II (TGF βR2) in CAR-T cells and found that inhibition of TGFβR2 prevented CAR-T exhaustion in preclinical hepatic and pancreatic tumor models [34].

Two innovative strategies explored to impede T cell exhaustion in the hostile TME include epigenetic and transcriptional modifications. Because DNA methylation is a central mediator in T cell differentiation and potency, a group created DNA methyltransferase 3a (DNMT3A) KO CAR-T cells. They aimed to investigate if the cells’ preserved functional capacity against chronic tumors and rechallenge experiments in both in vitro and NSG murine solid tumor models [35, 36•]. Not only did the DNMT3A-KO CAR-T cells retain proliferative capacity, but the cells also controlled a secondary tumor challenge, conserving memory potential and protecting against tumor relapse. The study identified DNMT3A exhaustion signature as a potential biomarker for future studies. In attempts to address the hypoxic environment of TME, hypoxia-inducible CARs (HiCARs) have been modified to include hypoxia response elements (HRE) and an oxygen-dependent degradation domain (ODD) that degrades under normal environments but remain stable under low-oxygen conditions [37]. ODD fusion was discovered to achieve oxygen-dependent activation of CAR-T cells and maintain effector T cell function.

### Safety Control: On-target, Off-tumor toxicity, and Cytokine Release Syndrome (CRS)

On-target off-tumor toxicity can be detrimental when CAR-T cells are directed to attack a tumor antigen that is also present in normal tissues. For example, low-level HER2 expression on lung epithelia most likely contributed to a fatal case report in a HER2-CAR-T clinical trial [38]. To avoid these effects, modulating the scFV has been tested. In a murine model, the affinity of GD2-CAR-T cells was varied, causing different levels of neurotoxicity [39]. Various alterations have been tested to optimize this strategy, like integrating the synthetic Notch (synNotch) system, a receptor based on the “prime-and-kill” strategy that recognizes antigen A presented on tumor cells which trigger the expression of a CAR-attacking tumor antigen B [40, 41]. Through this “AND” logic gate, only the synNotch receptor with a low-affinity scFv could achieve ultrasensitivity and spare normal cells in in vitro assays of diverse HER2 tumor densities. While modulating the scFv has shown promise, this strategy is ideal when antigens are expressed at very high levels and does not nullify the potential for tumor relapse. Thus, additional investigation is warranted.

On–off switches are one novel strategy based on drug-controlled regulation of CAR signaling that allows physicians to alter CAR-T activity after infusion to mitigate cytokine release syndrome (CRS) toxicity. One group demonstrated that the tyrosine kinase inhibitor, dasatinib, interfered with the lymphocyte-specific protein tyrosine kinase (LCK) and thereby ablated signaling in CAR constructs [42]. Depending on the dosing regimen, they showed that dasatinib could partially or entirely inhibit CAR-T cell function. Furthermore, dasatinib administration after CAR-T cell infusion protected a proportion of mice from CRS in their lymphoma xenograft mouse model. Another group engineered small protein tags onto CD-19 (4-1BB) CAR-T cells that would be vulnerable when treated with FDA approved drug lenalidomide, commonly used in multiple myeloma, which induces proteasomal breakdown of several target proteins through modulation of CRL4^CRBN^ E3 ubiquitin ligase [43]. Through the administration of lenalidomide, the off switch, CAR-T treatment was paused temporarily, preventing toxicities. When lenalidomide was stopped, CAR-T cells switched back on and resumed their anti-tumor activity. While this study focused on lymphoma cells, its results are promising for application in solid tumor models.

Another exciting approach for mitigating toxicities is the integration of suicide genes through inclusion of the inducible caspase 9 (iC9) in CAR constructs to trigger apoptosis and control CAR-T cell activity [42, 43].

## CAR-T Cells for Pediatric Solid Tumors—A Clinical Experience

### Pediatric Brain Tumors (PBTs)

Brain tumors represent the most common and lethal solid cancer in the pediatric oncological population. These neoplasms are heterogeneous, arising from glial or neuronal cells, and include gliomas and embryonal tumors such as, medulloblastomas and ependymomas [44]. The incidence of pediatric brain tumors (PBTs) varies globally, but the USA reports the highest rates, with 1.15 to 5.14 cases per 100,000 children [45]. Many factors influence prognosis, including age, tumor grade, anatomical site, histological type, and demographics. Over the last four decades, substantive progress has increased the 5-year survival rate from 55 to 76%; however, about one-third of PBT survivors have reduced quality of life and suffer neurocognitive decline from aggressive standard treatment [46]. Adverse long-term sequelae and low survival rates for rare brain tumor subtypes unveil the critical need for the development of alternative therapy.

#### Tumor Targets

Five tumor-associated antigens have been identified in PBTs, including B7 homolog 3 protein (B7-H3), epidermal growth factor receptor (EGFR), Herceptin-2 (HER2), disialoganglioside 2 (GD2), and IL-13 Rα2. All are in clinical testing (NCT04510051, NCT04185038, NCT03638167, NCT04099797, NCT03500991). In February 2022, Majzner et al. published early results from their phase I dose-escalation trial of GD2-CAR-T cells (4-1BB costimulatory domain and a CD3 ζ signaling domain) for children and young adults with histone 3 K27M (H3K27M) mutant pontine and spinal cord DMGs (NCT04196413) [47•]. Four patients (one with spinal DMG) received 1 × 10^6^ GD2-CAR-T cells per kilogram administered intravenously, and three patients experienced radiographic and clinical improvement after I.V. infusion. These patients then received escalated doses of GD2-CAR-T cells through intracerebroventricular (I.C.V.) administration. All patients who underwent I.C.V. infusions of CAR-T cells gained additional radiographic or clinical benefits and encountered less systemic toxicity than I.V. administration. While all patients, unfortunately, passed away, three patients lived ≥ 20 months after diagnosis, double the amount of time for DMGs patients with an average life expectancy of 10 months after diagnosis [48]. Although this early report includes a small pool of patients, the clinical benefits observed are promising. The trial is ongoing to determine the optimal dosage regimen, route of delivery, and safety of GD2-CAR-T cells.

Interim analysis of another clinical trial involving three patients (age range 16–26 years old) with CNS tumors that received six to nine locoregional doses of HER-2-CAR-T cells demonstrated clinical evidence of feasibility and safety (NCT03500991) [49]. While early, results have not shown radiographic tumor regression. Furthermore, according to Vitanza et al., this is the first clinical trial to report results with repetitive locoregional dosing of HER-2 CAR-T cells in younger patients with CNS tumors. The team has initiated clinical trials investigating EGFR-, B7H7-, and HER2-CAR-T cells for pediatric CNS tumors and is using this experience to develop multiplexed strategies to overcome challenges such as tumor heterogeneity and confirm feasibility (NCT03638167, NCT04185038, NCT01109095) [50].

#### Future Directions

Given the heterogeneity of antigen expression in brain tumors, bi-specific and multivalent CAR-T cells have been designed [18, 51]. Trivalent CAR-T cells targeting IL13Rα2, HER2, and EphA2 were tested in a GBM orthoptic xenogeneic mouse model through intratumoral administration [52]. The trivalent CAR-T cells killed 100% of tumor cells in nearly all models tested. This construct also showed efficacy in preclinical studies for recurrent and metastatic medulloblastoma and ependymoma when delivered to the cerebrospinal fluid [53]. While a previous pilot study showed evidence of efficacy and tolerability of a peptide-based vaccine targeting these three targets, it was only effective in human leukocyte antigen (HLA)-A2 positive patients [54]. The multivalent CAR-T cell is exciting because it is not limited by HLA restriction and can apply to a broader patient pool for malignant CNS tumors. However, the trivalent product has not been translated to a clinical trial, as researchers intend to include modifications to enhance efficacy, such as adding a signaling domain or safety mechanism to the CAR construct.

### Neuroblastoma

Neuroblastoma (NB), the most common extracranial cancer in children, is a neoplasm of the sympathetic nervous system deriving from neural crest cells that typically originate in the adrenal medulla and paraspinal ganglia [55]. Over 90% of cases occur in children < 5 years with variable clinical presentation, involving spontaneous regression of localized tumors, intermediate-risk tumors treated through surgical resection with or without adjuvant treatment, and high-risk disease with often metastatic disease and/or N-MYC gene amplification [56]. Improvements in multimodal therapies have increased 5-year survival rates from < 20 to < 50% over the past decades for patients with high-risk neuroblastoma. Nonetheless, high-risk cases of NB pose a constant challenge in pediatric oncology, and the particular focus is on GD2 to advance outcomes for these vulnerable pediatric patients.

#### Tumor Targets

Although the adhesion molecule L1-CAM has been targeted for neuroblastoma in both preclinical and clinical studies, GD2 is unquestionably the leading antigenic target investigated for the development of CAR-T cells due to its high expression in the tumor [57]. Multiple clinical trials are underway using second- and third-generation GD2-specific CARs incorporating different signaling costimulatory molecules into the construct (Table 1). While these experiences thus far have confirmed the safety and feasibility of various GD2-CAR products, limited T cell persistence appears to be a critical obstacle to success. Recently, results from a multi-institutional phase II trial using fourth-generation GD2-CARs (including additional CD28, 4-1BB, and CD27 costimulatory domains) modified with the iC9 suicide gene have been reported [58•]. After receiving fludarabine and cyclophosphamide (Flu/Cy) for lymphodepletion, ten patients with refractory or recurrent high-risk neuroblastoma received one or more intravenous infusions of 4SCAR-GD2 T cells. Notably, safety and feasibility were established for the newly developed 4SCAR-GD2 T cells as no patients required intervention for neurotoxicity events, and the CAR-T cells persisted for more than 6 months. Interestingly, CAR-T cells expanded when the disease relapsed past 6 months, suggesting that the tumor re-recurrence could stimulate this CAR construct. Due to a limited patient pool, no correlation between infusion dose and T cell expansion was found, so optimal dosage of CAR-T cells is not known. Further research is warranted to establish efficacy. Researchers plan to incorporate combinatorial strategies, such as including a PD-1 inhibitor or an oncolytic virus, for further study with 4SCAR-GD2 T cells.

**Table 1:** Ongoing clinical trials using chimeric antigen receptor T cells (CAR-T) for pediatric solid tumors

NCT number	Title	Status	Conditions	Locations
NCT02932956	Glypican 3-specific chimeric antigen receptor expressed in t cells for patients with pediatric solid tumors (GAP)	Active, not recruiting	Liver cancer	Texas Children’s Hospital, Houston, TX, USA
NCT04377932	Interleukin-15 armored glypican 3-specific chimeric antigen receptor expressed in t cells for pediatric solid tumors	Recruiting	Liver cancer|rhabdomyosarcoma|malignant rhabdoid tumor|liposarcoma|Wilms tumor|yolk sac tumor	Texas Children’s Hospital, Houston, TX, USA
NCT04715191	Interleukin-15 and interleukin-21 armored glypican-3-specific chimeric antigen receptor expressed in t cells for pediatric solid tumors	Not yet recruiting	Liver cancer|rhabdomyosarcoma|malignant rhabdoid tumor|liposarcoma|Wilms tumor|yolk sac tumor	Texas Children’s Hospital, Houston, TX, USA
NCT04897321	B7-H3-specific chimeric antigen receptor autologous T cell therapy for pediatric patients with solid tumors (3CAR)	Recruiting	Pediatric solid tumor|osteosarcoma|rhabdomyosarcoma|neuroblastoma|Ewing sarcoma|Wilms tumor|adrenocortical cancer|desmoplastic small round cell tumor|germ cell cancer|rhabdoid tumor|clear cell sarcoma|hepatoblastoma|melanoma|carcinoma|malignant peripheral nerve sheath tumors|soft tissue sarcoma	St. Jude Children’s Research Hospital, Memphis, TN, USA
NCT04483778	B7H3 CAR T cell immunotherapy for recurrent/refractory solid tumors in children and young adults	Recruiting	Pediatric solid tumor|germ cell tumor|retinoblastoma|hepatoblastoma|Wilms tumor|rhabdoid tumor|carcinoma|osteosarcoma|Ewing sarcoma|rhabdomyosarcoma|synovial sarcoma|clear cell sarcoma|malignant peripheral nerve sheath tumors|desmoplastic small round cell tumor|soft tissue sarcoma|neuroblastoma|melanoma	Seattle Children’s Hospital, Seattle, WA, USA
NCT03618381	EGFR806 CAR T cell immunotherapy for recurrent/refractory solid tumors in children and young adults	Recruiting	Pediatric solid tumor|germ cell tumor|retinoblastoma|hepatoblastoma|Wilms tumor|rhabdoid tumor|carcinoma|osteosarcoma|Ewing sarcoma|rhabdomyosarcoma|synovial sarcoma|clear cell sarcoma|malignant peripheral nerve sheath tumors|desmoplastic small round cell tumor|soft tissue sarcoma|neuroblastoma	Seattle Children’s Hospital, Seattle, WA, USA
NCT03373097	Anti-GD2 CAR T cells in pediatric patients affected by high risk and/or relapsed/refractory neuroblastoma or other GD2-positive solid tumors	Recruiting	Neuroblastoma|neuroblastoma recurrent|GD2-positive solid tumors|osteosarcoma|Ewing sarcoma|sarcoma	Ospedale Pediatrico Bambino GesÃ^1^, Roma, Italy

Table data generated October 17, 2022, from ClinicalTrials.gov

Other groups have attempted to modify GD2-CAR constructs with different receptors and cytokines to enhance T cell persistence. For example, results from a phase I trial of second-generation (CD28/CD3ζ) anti-GD2 CARS in London utilizing an scFv based on humanized murine antibody KM8138 have been published (NCT02761915) [59]. Straathof et al. escalated both cell dose and preparative lymphodepletion regimen before 12 patients received intravenous infusions of the T cells. Three of the six patients who received the highest doses demonstrated regression of disease. Furthermore, in all patients, no severe neurotoxicity occurred. Ultimately, no patients had objective clinical responses, but the study’s foremost consideration was safety, and it was accomplished. Investigators hope to incorporate next-generation approaches in the trial from these mixed results. Another ongoing clinical trial at Baylor College of Medicine investigated GD2-CAR-T cells modified with cytokine interleukin-7 (IL-7) to increase T cell survival for neuroblastoma (NCT03635632). The study’s primary completion date is estimated to be in June 2023.

#### Future Directions of Neuroblastoma

Last year, a team published a study attempting to delineate a mechanism of resistance using their optimized GD2-specific CAR constructs currently under evaluation in phase I/Il clinical trial for patients with relapsed and refractory NB (Table 1) (NCT03373097) [60]. In NB patients who relapsed or did not respond to GD2-CAR-T cell treatment, polymorphonuclear myeloid–derived suppressor cells (PMN-MDSC) were increased in the peripheral blood (PB). PMN-MDSCs were then isolated and subjected to in vitro analysis, where researchers confirmed the strong inhibitory capability of these suppressor cells on different generations of GD2-CAR constructs. Interestingly, in NB patients who had undergone GD2-CAR-T treatment, the prevalence of PMN-MSDC is inversely correlated with the levels of GD2-CAR T cells, resulting higher in patients who did not respond or began failing to treatment. Because of the study, PMN-MDSCs have emerged as potential targets and biomarkers for further optimization. Similarly, the use of naxitamab, a humanized anti-GD2 monoclonal antibody, and sargramotism (GM-CSF) are being explored as treatment options for high-risk neuroblastoma patients who experience incomplete remission [61].

Glypican-2 (GPC2), an oncofetal target expressed in early development that regulates cell growth and is limited in postnatal human tissues, was more recently discovered to be overexpressed in neuroblastoma [62–64]. As such, studies are emerging that investigate on GPC2-targeted CAR-T cells for NB. Researchers used NB clinical samples in one study to measure GPC2 antigen density in pediatric bone marrow specimens. They discovered that GPC2 antigen density was below the threshold required for anti-tumor efficacy in traditional GPC2-CAR-T cells and modified them using CD28 co-stimulatory endodomains. The attuned GPC2-CAR mediated potent activity without toxicity in vivo NB models. Another study used microRNA (mRNA) to introduce GPC2-CARs into T cells, a derivation from the traditional method that most often utilizes viral vectors [65•]. The cytotoxicity of mRNA GPC2-CAR-T cells proved successful in NB cell lines. Further investigations are needed to measure in vivo durability of these CAR constructs as well as the effects of repeated dosing.

### Sarcoma

Sarcomas are a group of rare, highly heterogeneous connective tissue cancers such as Ewing sarcoma (EWS) and rhabdomyosarcoma (RMS), with the latter accounting for more than 50% of pediatric sarcoma tumors [66]. From a putative mesenchymal origin, sarcomas are vastly soft tissue malignancies often forming in the body’s muscles, fat, or nerves but also include malignant bone tumors and contribute to less than 1% of all solid cancers in adults [67].

Fortunately, most pediatric patients with localized sarcomas become long-term survivors due to chemotherapy, surgery, and radiotherapy. However, like in PBT cases, minimizing treatment-related morbidity is a continuous goal. Unfortunately, approximately half of adult survivors of RMS suffer from RT-associated chronic health conditions [68].

#### Tumor Targets

Several CAR-T cell clinical trials are in progress for children with different sarcoma subtypes (Table 1). HER2 emerged as a leading target due to its expression in multiple subtypes, thus driving numerous trials for pediatric sarcomas [69–72]. One group attempted to improve their previous dose-escalation study of HER2 CAR-T cells by using a combination of Cy/Flu lymphodepletion before HER2 CAR-T administration in children with refractory or metastatic osteosarcoma, RMS, and synovial sarcoma (NCT00902044). A new report on the trial described tumor remission in a child with metastatic RMS after HER2 CAR-T cell infusion combined with checkpoint inhibitor pembrolizumab [73]. Endogenous immune reactivity was also observed, and by the publication date, the child had a complete response 20 months after the last CAR-T cell infusion. If the results are reproducible, treatment holds significant promise in a small number of sarcoma patients who fail to respond to standard therapy and, thus, bear a grim prognosis.

New York esophageal squamous cell carcinoma-1 (NY-ESO-1) is highly expressed in synovial sarcomas and myxoid liposarcomas and has also shown promising results in clinical trials. The most recent completed phase I trial tested NY-ESO-1 CAR-T cells in patients with low and high NY-ESO-1–expressed synovial sarcomas after lymphodepletion using a combination of Cy/Flu or Cy only (NCT01343043) [74, 75]. Patients with clinical response to the therapy for > 3 months received a second administration of NY-ESO CAR-T cells. After infusion, one patient achieved a complete response, while 14 demonstrated a partial response.

While CAR-T clinical trials have garnered some clinical benefits, most patients inevitably experience tumor relapse. To combat this, researchers have attempted to modify T cells to activate MyD99, the central signaling hub for the innate immune system, to sustain CAR-T function after re-exposure to tumor cells [76]. One group created an autocrine loop using cytokine receptor GM18 that binds GM-CSF, a cytokine-dependent upon CAR-T activation, to IL-18 receptor signaling, which operates through MyD99 [77]. The GM18 receptor was expressed in second-generation CAR-T cells targeting EphA2 and HER2 in osteosarcoma and EWS tumor models. Promisingly, 90% of tumors shrank, and repeated stimulation assays demonstrated no significant decrease in GM-CSF production, proving that the loop remained intact. Furthermore, these GM18-modified T cells induced tumor regression at lower cell doses than standard CAR-T cells. The results of this study present a potential approach to improve current T cell strategies for pediatric sarcoma.

#### Future Directions

Numerous encouraging preclinical studies have tested new CAR-T targets against in vitro and in vivo models of pediatric sarcoma. These latest markers include fibroblast growth factor receptor 1 protein (FGFR1), platelet-derived growth factor receptor alpha (PDGFRA), and vascular endothelial growth factor receptor 2 (VEGFR2) [69, 78–82]. These targets are investigated for clinical development, and prospects will focus on patient safety and efficacy.

## Conclusion

The therapeutic benefit of CAR-T cell therapy, as observed in hematological malignancies, has not been translated into solid cancers—obstacles such as heterogeneous antigen presentation and evasion of immune surveillance challenge this success in solid tumors. Over the past decades, several target antigens have been identified for various solid tumors, and some have shown safety and feasibility in preclinical and clinical studies. Despite ongoing research, none of these therapies have been approved for clinical application, and many are still far from it. These failures highlight that effective treatment of solid tumors may depend on combination therapies, with CAR-T cells targeting multiple antigens or modified to include safety switches. Future efforts should continue to focus on combinatorial strategies to combat the immunosuppressive TME and design dosing schedules and delivery methods to enhance CAR-T cell application best. Furthermore, close attention to the safety of these therapies is especially needed in the scope of the pediatric population.
